# Supplementary material for: Patient-derived lung cancer organoids as in vitro cancer models for therapeutic screening
Source: Nat Commun. 2019 Sep 5;10:3991. doi: 10.1038/s41467-019-11867-6 (PMC6728380; doi:10.1038/s41467-019-11867-6)
Supplement: Supplementary file 1 — Supplementary Information [file 41467_2019_11867_MOESM1_ESM.pdf]

Supplementary information

Patient-Derived Lung Cancer Organoids as *In Vitro* Cancer Models for Therapeutic Screening

Kim et al.

Supplementary Methods

LCO culture from needle biopsy tissue. A needle biopsy specimen (~ 1 x 30 mm size) from a patient with advanced lung adenocarcinoma was obtained. The biopsy tissue was incubated with 0.001% DNase (Sigma-Aldrich, MO, USA), 1 mg/ml collagenase/dispase (Roche, IN, USA), 200 U/ml penicillin, 200 mg/ml streptomycin, and 0.5 mg/ml amphotericin B (2% antibiotics, Sigma) in DMEM/F12 medium (Lonza, Basel, Switzerland) at 37°C for 1 h with intermittent agitation. After incubation, the suspensions were repeatedly triturated by pipetting. The cells were centrifuged at 112 *g* for 3 min, and the pellet was resuspended in 50 µl MBM (serum-free medium (DMEM/F12; Lonza) supplemented with 20 ng/ml of basic fibroblast growth factor (Invitrogen, CA, USA), 50 ng/ml human epidermal growth factor (Invitrogen), N2 (Invitrogen), B27 (Invitrogen), 10 µM ROCK inhibitor (Enzo Life Sciences, NY, USA), and 1% penicillin/streptomycin (Gibco, OK, USA)) and 100 µl Matrigel. The resulting cell suspension was allowed to solidify on pre-warmed 6-well culture plates (Corning, NY, USA) at 37°C for 10 min. After gelation, 3 ml MBM was added to the well. The medium was changed every 4 days.

Statistical analysis. Data are mainly expressed as a mean ± SEM from at least three experiments. Statistical analysis was performed with SPSS and graphs were designed using Graph Pad Prism 5. $P < 0.05$ was considered to indicate a statistically significant difference.

Supplementary Figure Legends

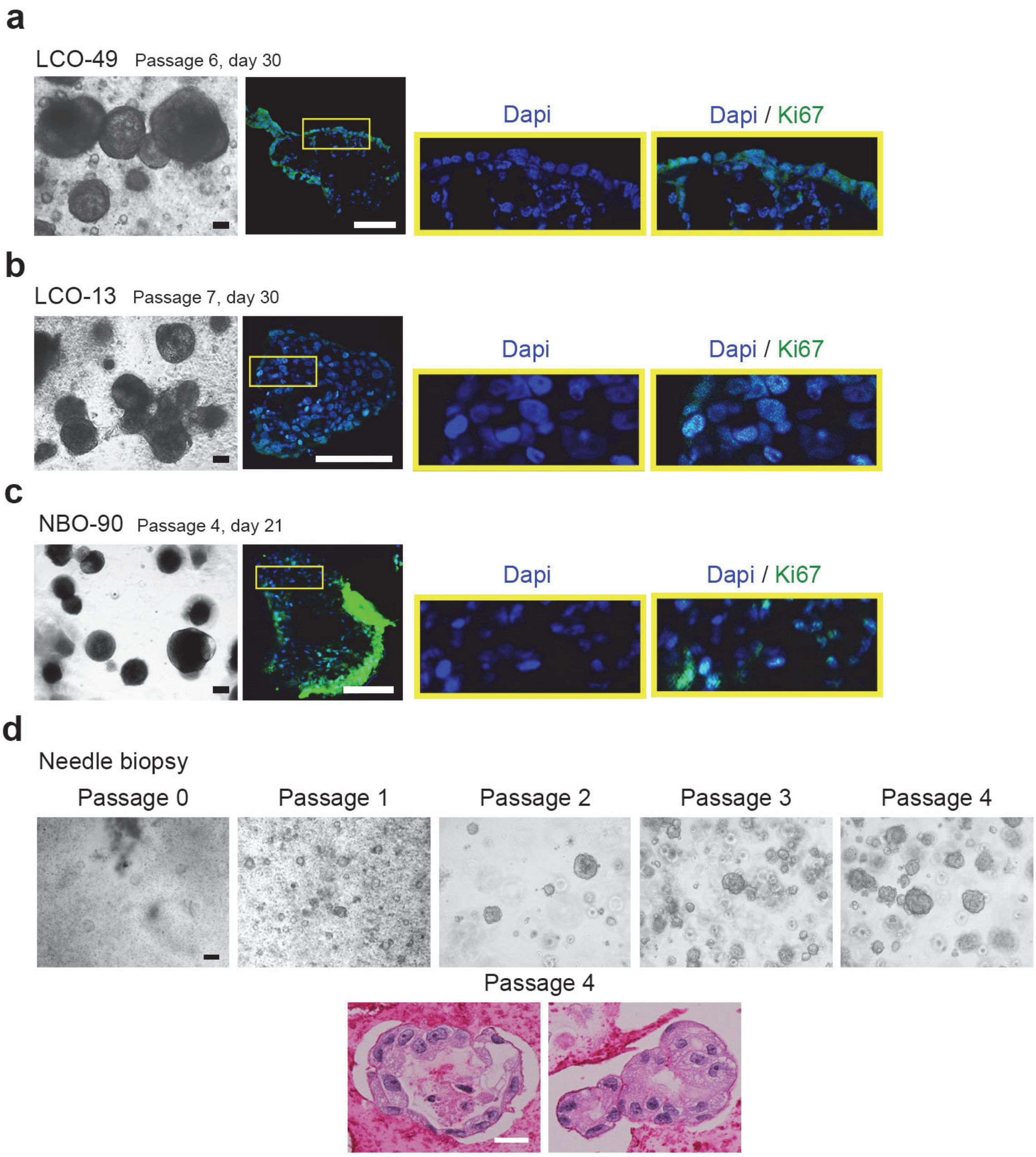

Supplementary Figure 1. LCOs were generated from resected fresh tissues and needle biopsy tissue. (a-c) Bright-field microscopy and immunofluorescence images showing the growth and proliferation of long-term cultured organoids. These organoids were generated from cancer or normal tissues of patients. Proliferating cells in organoids were stained with Ki67 and Nuclei (blue) were stained with DAPI. Scale bar, 100 μm. The information of organoids in these images; LCO-49; adenocarcinoma, LCO-13; squamous cell carcinoma, NBO-90; normal bronchus. (d) Bright-field microscopy and H&E

stained images showing the growth of adenocarcinoma organoid from a needle biopsy sample. Scale bar in bright-field microscopy images, 100 μm. Scale bar in H&E staining images, 20 μm.

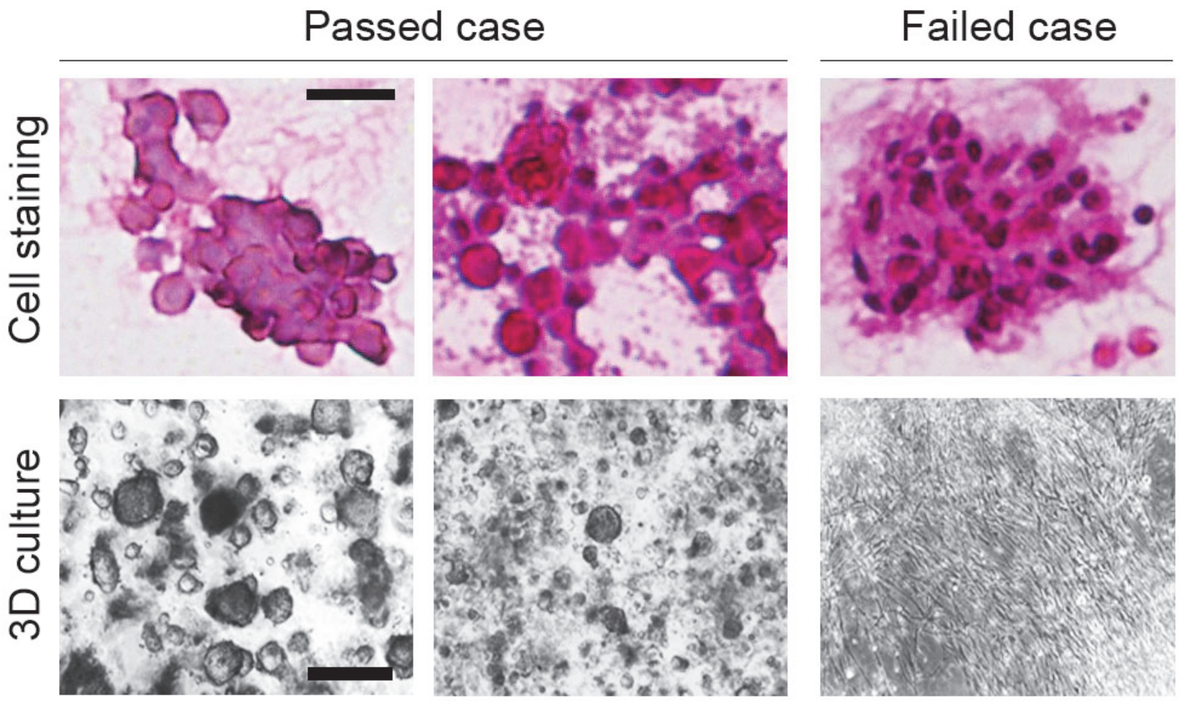

Supplementary Figure 2. Cellular composition of LC tissues affected successful formation of LCOs. H&E images of cells from parental LC tissues (upper row) and bright-field microscopy images of LCOs from the LC tissues (bottom row). LC tissues with epithelial-like cells (round shape) as main component were passed the initial cytologic quality evaluation (Passed case) and they were successfully formed in 3D culture condition. In contrast, LC tissues with fibroblast-like cells (spindle shape) as major component (Failed case) failed to form LCOs. Scale bar, 50 μm (upper row), 100 μm (bottom row).

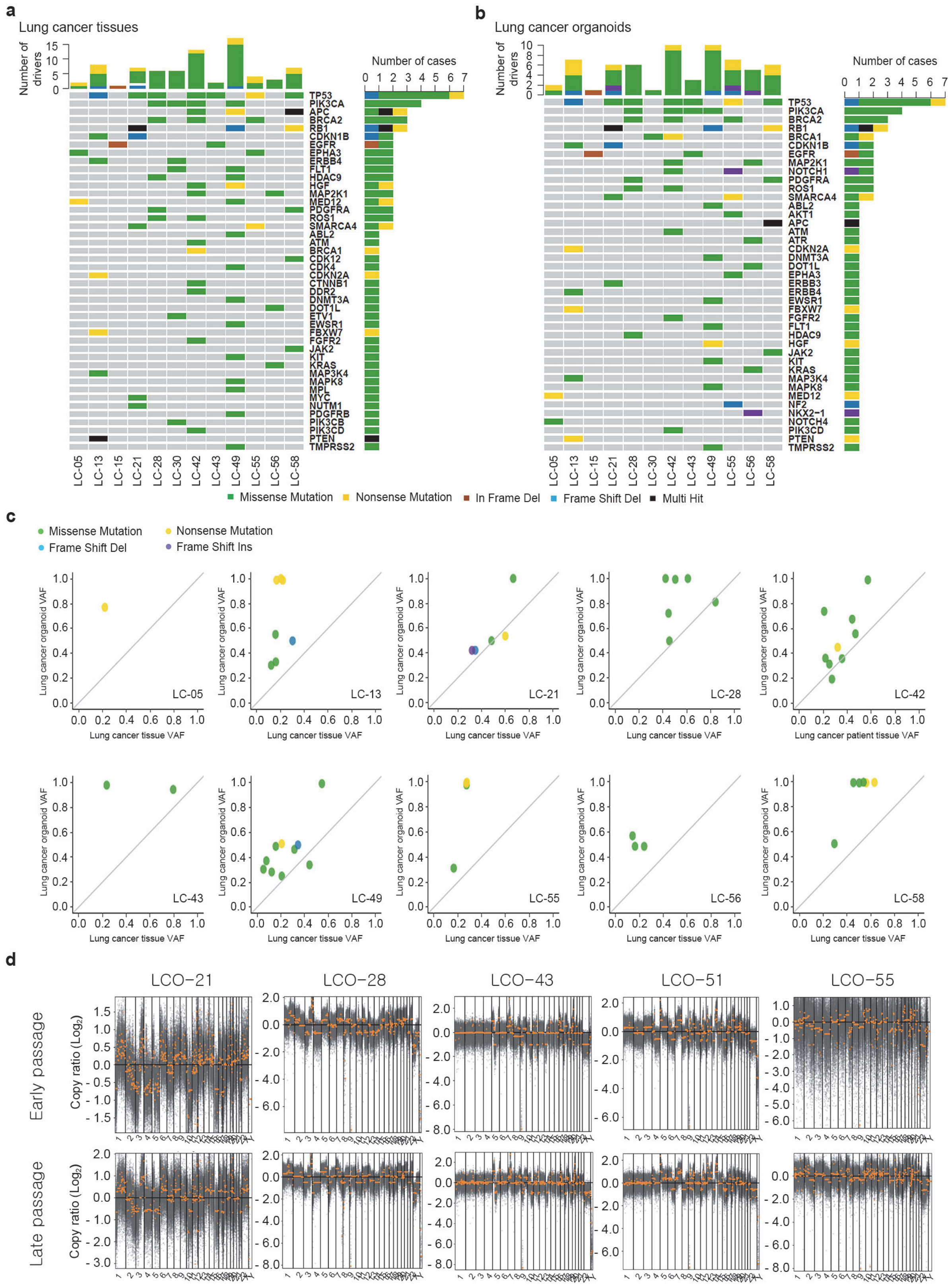

Supplementary Figure 3. Oncoplot summary of mutated genes in 12 paired LC tissues and LCO and copy number variation data compared with organoids in early and late passage. (a, b) The histogram

shows the number of genetic alterations that were observed in each LC tissue and organoid. The histogram on the right in each panel shows the number of genetic alterations that were observed in individual driver genes. In 12 LC tissues, mutated 44 of captured total 203 genes including 164 cancer-related genes were detected (a). On the other hand, mutated 41 of captured total 203 genes were detected in 12 LCOs (b). (c) The comparison of VAF of genetic alterations detected in LC tissue and organoid in individual case. (d) Whole-genome profiles of $\log_2$ copy ratio by CNVkit (Copy number variation kit) in 5 pairs of LCOs in early and late passage.

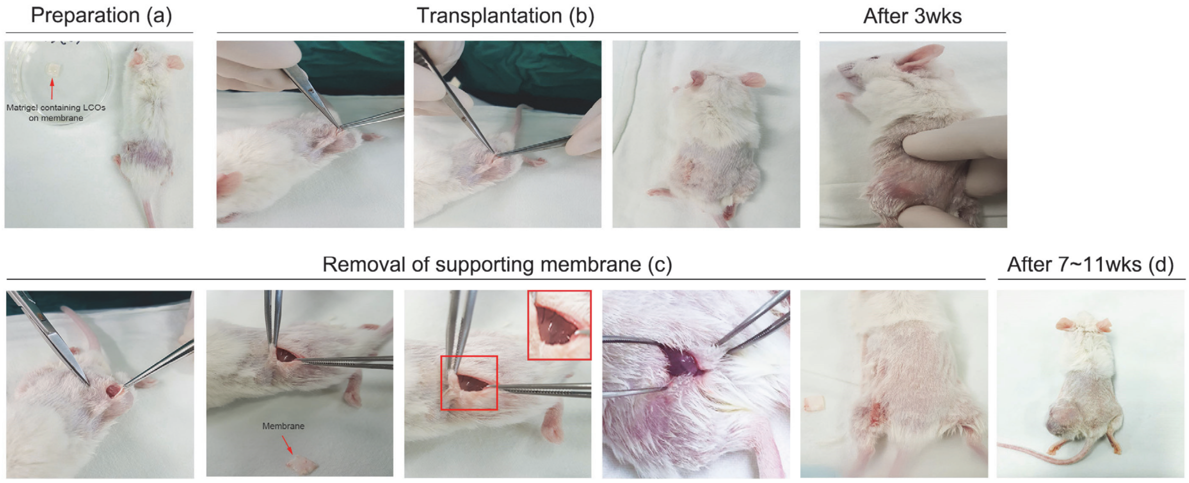

Supplementary Figure 4. Modified xenograft method maintaining the 3D structure of LCOs. (a) LCOs were prepared as a Matrigel drop on a supporting membrane. (b) The membrane including LCOs was transplanted into a mouse. (c) After 3 weeks, the membrane was removed from the mouse. Tumour formation and growth of blood vessels around the tumour were visible. The enlarged image showed blood vessels around tumour. (d) After 7 to 11 weeks, grossly visible tumour formation was confirmed.

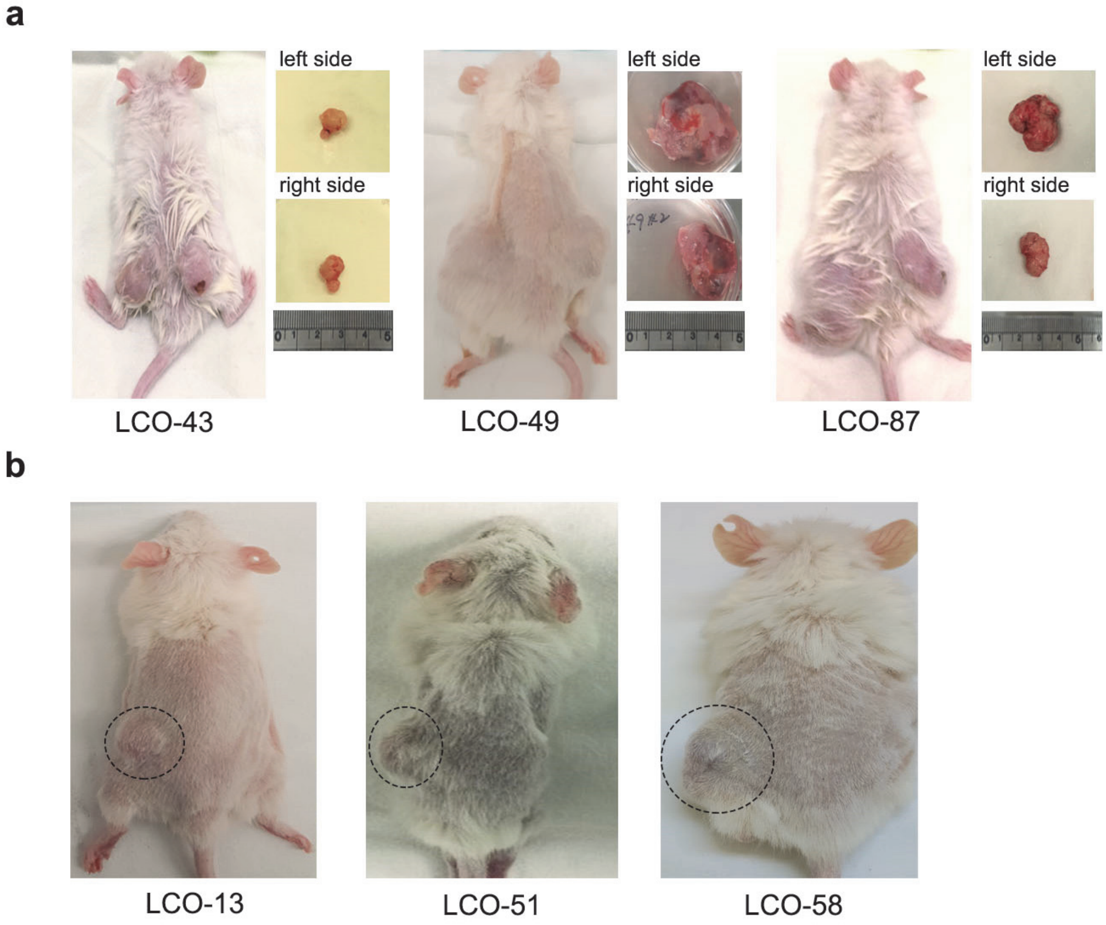

Supplementary Figure 5. LCOs maintaining 3D structure (left buttock) generated tumours in mice more efficiently than dissociated LCO cells (right buttock). (a) Both sides formed tumours, however, sizes of left side tumours are similar (LCO-43) or bigger (LCO-49 and LCO-87) than right side tumours. (b) LCO-13, LCO-51 and LCO-58 formed tumours only in left side buttocks implanted by 3D organoids.

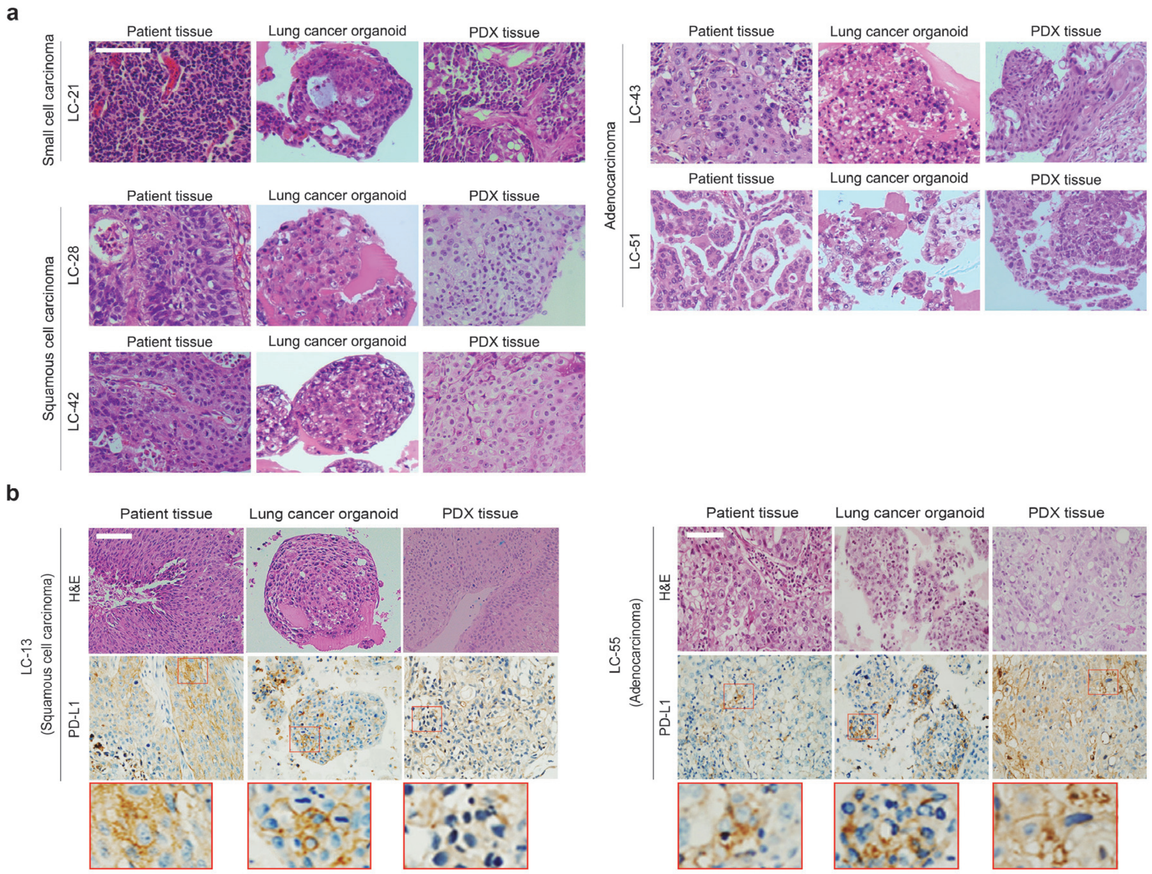

Supplementary Figure 6. LCOs as well as PDX tumours from the LCOs maintained the characteristics of their parental lung cancer. (a) Comparative H&E stained images of patient tissues, LCOs, and PDX tumours from the LCOs. Scale bar, 100 μm. (b) Comparative H&E and PD-L1 stained images of patient tissues, LCOs, and PDX tissues. The expression of PD-L1 in patient tissue is maintained LCOs and PDXs. Scale bar, 100 μm. Panels below show enlarged images of the boxed areas.

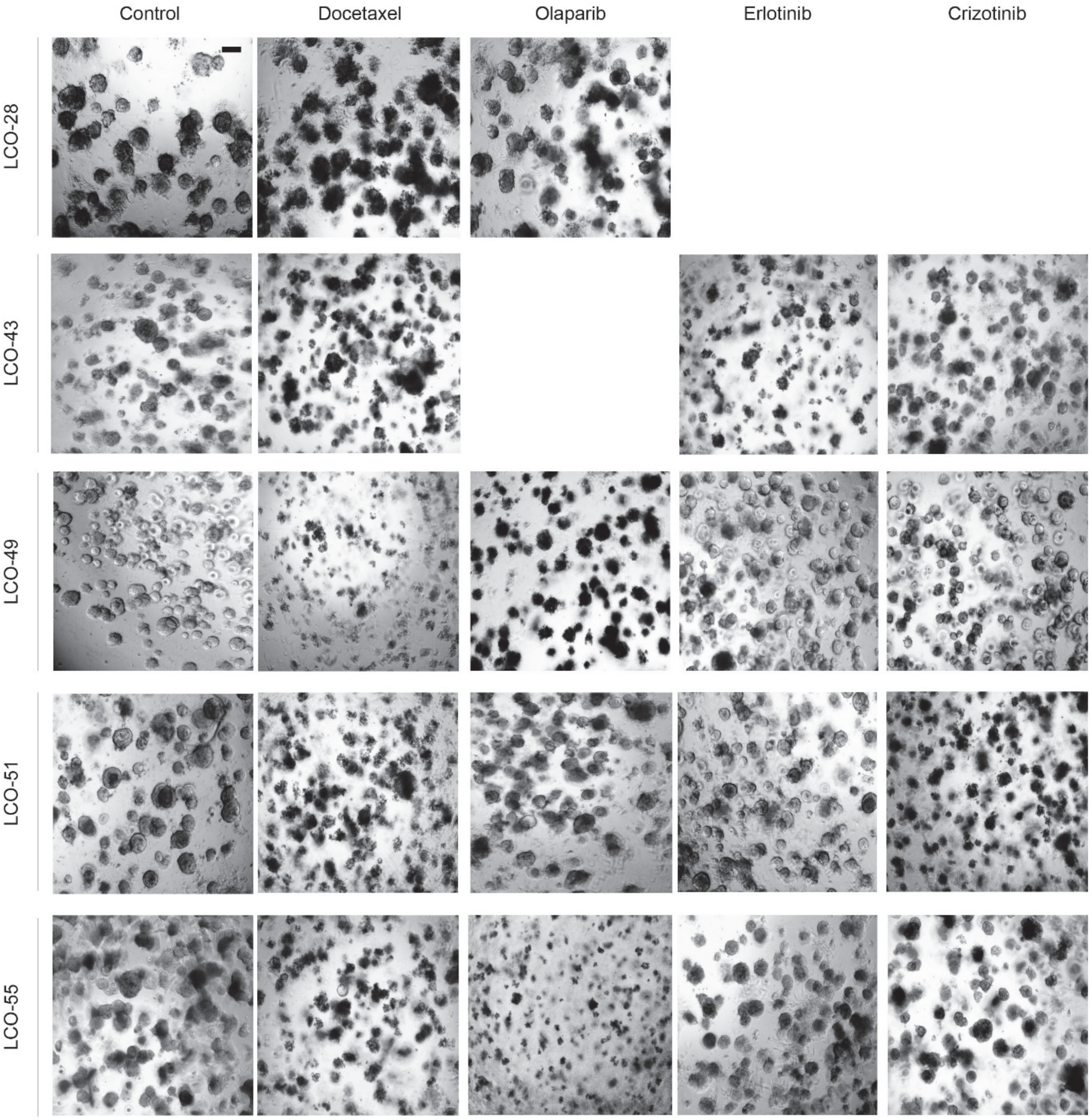

Supplementary Figure 7. Each LCOs responded differently to anti-cancer drugs. Bright-field microscopy images showing the sizes and morphological changes of LCOs following treatment with 100 nM anti-cancer drugs. Scale bar, 100 μm.

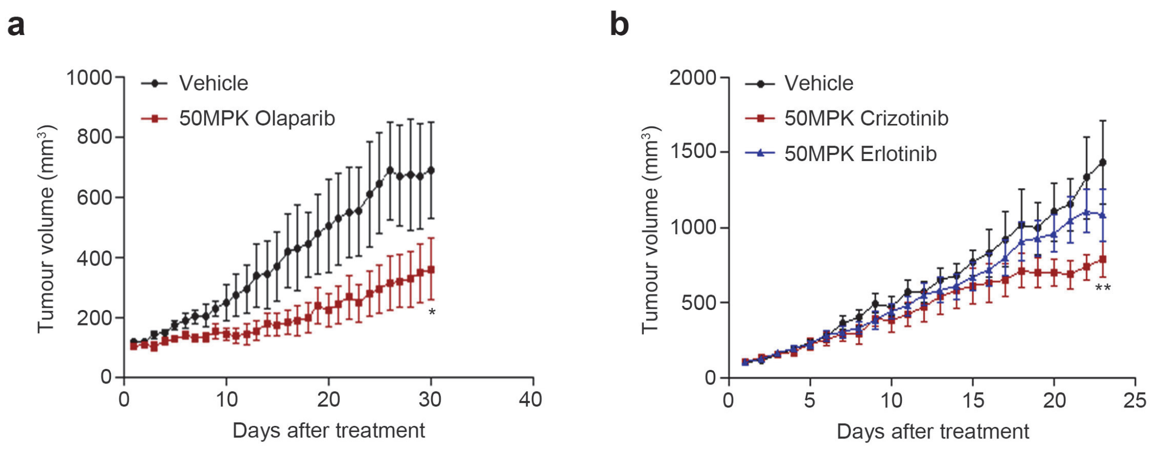

Supplementary Figure 8. Drug responses of *in vivo* model generated from LCOs. (a) The tumour growth curve of subcutaneous xenografts generated from LCO-55 adenocarcinoma after treatment of olaparib and vehicle only. (p-value was determined by paired t-test). (b) The tumour growth curve of xenografts generated from LCO-51 adenocarcinoma after treatment of 50 MPK crizotinib, erlotinib and vehicle only (p-value was determined by one-way ANOVA). All drugs were treated for the indicated period as soon as tumour volumes reached 80 ~ 120 mm^3.

All error bars indicate SEM, n = 4. *, $P < 0.05$; **, $P < 0.01$.

Figure 6g. LCO-43

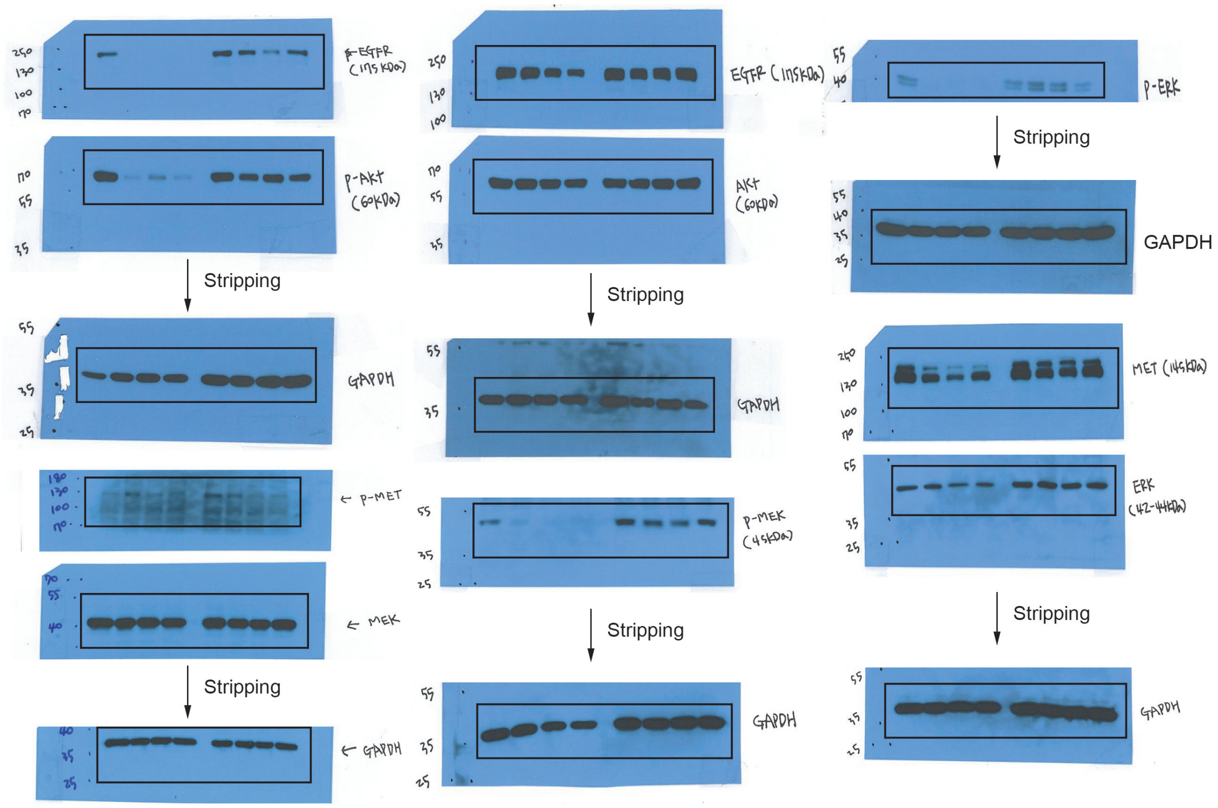

Figure 6g. LCO-51

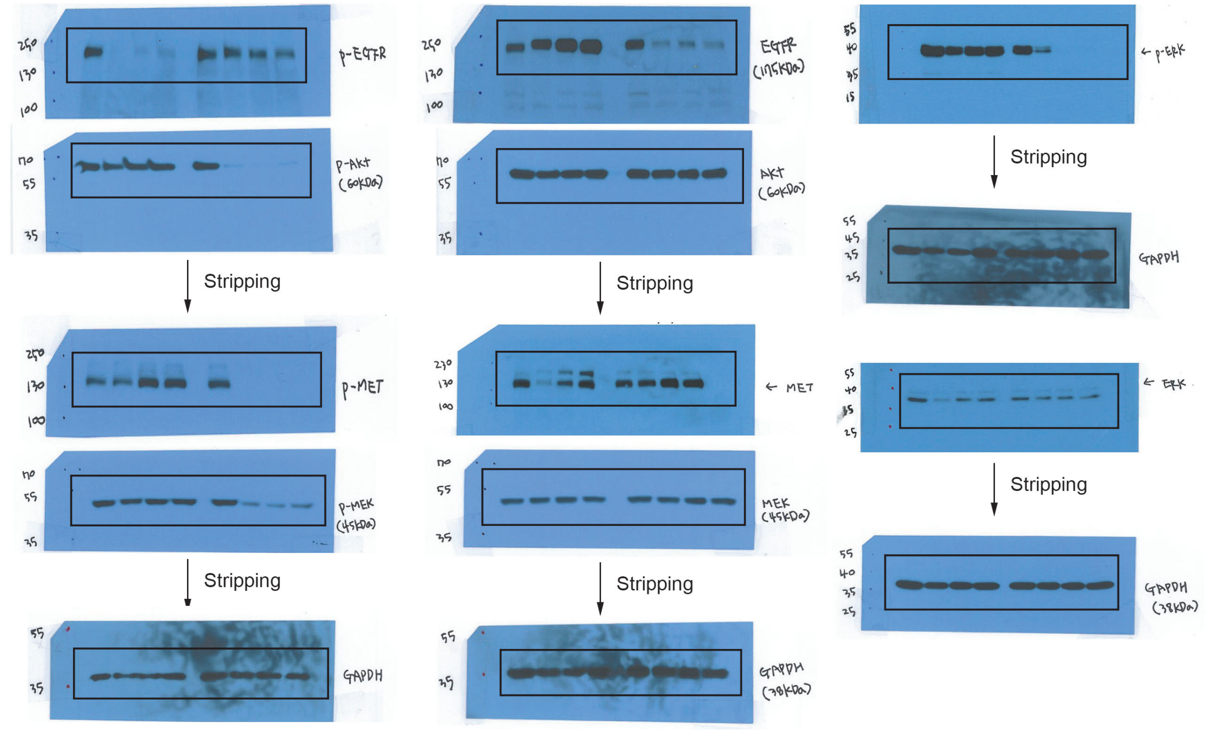

Supplementary Figure 9. Full-length blot in Figure 6.
