# Supplementary material for: Patient-derived lung cancer organoids as in vitro cancer models for therapeutic screening
Source: Nat Commun. 2019 Sep 5;10:3991. doi: 10.1038/s41467-019-11867-6 (PMC6728380; doi:10.1038/s41467-019-11867-6)
Supplement: Supplementary file 3 — Reporting Summary [file 41467_2019_11867_MOESM3_ESM.pdf]

nature research

Corresponding author(s): Se Jin Jang

Last updated by author(s): Jun 28, 2019

# Reporting Summary

Nature Research wishes to improve the reproducibility of the work that we publish. This form provides structure for consistency and transparency in reporting. For further information on Nature Research policies, see Authors & Referees and the Editorial Policy Checklist.

## Statistics

For all statistical analyses, confirm that the following items are present in the figure legend, table legend, main text, or Methods section.

| n/a | Confirmed | |
|---|---|---|
| ☐ | ☒ | The exact sample size (*n*) for each experimental group/condition, given as a discrete number and unit of measurement |
| ☐ | ☒ | A statement on whether measurements were taken from distinct samples or whether the same sample was measured repeatedly |
| ☐ | ☒ | The statistical test(s) used AND whether they are one- or two-sided<br>*Only common tests should be described solely by name; describe more complex techniques in the Methods section.* |
| ☐ | ☒ | A description of all covariates tested |
| ☐ | ☒ | A description of any assumptions or corrections, such as tests of normality and adjustment for multiple comparisons |
| ☐ | ☒ | A full description of the statistical parameters including central tendency (e.g. means) or other basic estimates (e.g. regression coefficient) AND variation (e.g. standard deviation) or associated estimates of uncertainty (e.g. confidence intervals) |
| ☒ | ☐ | For null hypothesis testing, the test statistic (e.g. *F*, *t*, *r*) with confidence intervals, effect sizes, degrees of freedom and *P* value noted<br>*Give P values as exact values whenever suitable.* |
| ☒ | ☐ | For Bayesian analysis, information on the choice of priors and Markov chain Monte Carlo settings |
| ☒ | ☐ | For hierarchical and complex designs, identification of the appropriate level for tests and full reporting of outcomes |
| ☒ | ☐ | Estimates of effect sizes (e.g. Cohen's *d*, Pearson's *r*), indicating how they were calculated |

*Our web collection on statistics for biologists contains articles on many of the points above.*

## Software and code

Policy information about availability of computer code

| | |
|---|---|
| Data collection | No software was used. |
| Data analysis | Genome Analysis Toolkit (GATK) (v1.6.5.), Burrows-Wheeler Aligner (v0.5.9), Picard package (v1.92), MuTect (v1.1.7), Variant Effect Predictor (v79), FastQC, Genomics Viewer (IGV), R software (v3.0), CNVkit, ZEN software (Zeiss), Excel for data assembly, Graph Pad Prism 5 (GraphPad Software) |

For manuscripts utilizing custom algorithms or software that are central to the research but not yet described in published literature, software must be made available to editors/reviewers. We strongly encourage code deposition in a community repository (e.g. GitHub). See the Nature Research guidelines for submitting code & software for further information.

## Data

Policy information about availability of data

All manuscripts must include a data availability statement. This statement should provide the following information, where applicable:

- Accession codes, unique identifiers, or web links for publicly available datasets
- A list of figures that have associated raw data
- A description of any restrictions on data availability

The complete datasets of the study are available from the corresponding author on reasonable request.

# Field-specific reporting

Please select the one below that is the best fit for your research. If you are not sure, read the appropriate sections before making your selection.

☒ Life sciences ☐ Behavioural & social sciences ☐ Ecological, evolutionary & environmental sciences

For a reference copy of the document with all sections, see nature.com/documents/nr-reporting-summary-flat.pdf

# Life sciences study design

All studies must disclose on these points even when the disclosure is negative.

| | |
|---|---|
| Sample size | For this experiments, sample size was not calculated. For initial quality check and developing culture methods, we subjected 36 lung cancer tissue samples. After developing the organoid culture methods, samples were subjected to organoid culture under the project of lung cancer organoid biobanking based on patients' consents. |
| Data exclusions | No data were excluded from the analyses. |
| Replication | Since we included detailed methods and sources of all reagents and protocols for experiments in the manuscript, lung cancer organoid culture can be easily reproduced. |
| Randomization | Out experiments is not statistical description but wet experiment. |
| Blinding | Blinding was not relevant to our study because we investigated information of all samples we got, which includes age, sex and histology |

# Reporting for specific materials, systems and methods

We require information from authors about some types of materials, experimental systems and methods used in many studies. Here, indicate whether each material, system or method listed is relevant to your study. If you are not sure if a list item applies to your research, read the appropriate section before selecting a response.

## Materials & experimental systems

| n/a | Involved in the study | |
|---|---|---|
| ☐ | ☒ | Antibodies |
| ☒ | ☐ | Eukaryotic cell lines |
| ☒ | ☐ | Palaeontology |
| ☐ | ☒ | Animals and other organisms |
| ☐ | ☒ | Human research participants |
| ☒ | ☐ | Clinical data |

## Methods

| n/a | Involved in the study | |
|---|---|---|
| ☒ | ☐ | ChIP-seq |
| ☒ | ☐ | Flow cytometry |
| ☒ | ☐ | MRI-based neuroimaging |

## Antibodies

| | |
|---|---|
| Antibodies used | Primary antibodies: histology and imaging_anti-napsin A (#NCL-L-Napsin A, Novocastra, IL, USA), anti-TTF-1 (#NCL-L-TTF-1, Novocastra), anti-cytokeratin 7 (CK7; #M7018, Dako, CA, USA), anti-p63 (#M731701-2, Dako), anti-cytokeratin 5/6 (CK5/6; # MA5-12429, Invitrogen), anti-CD133 (#64326, Cell Signaling Technology, MA, USA), anti-PD-L1 (#13684, Cell Signaling Technology), and anti-c-Met (#257261, Dako). Immunofluorescence_anti-pancytokeratin (Pan CK; #4545, Cell Signaling Technology), anti-p63 (#4892, Cell Signaling Technology), anti-mucin 1 (MUC1; #ab45167, Abcam, MA, USA), anti-CC10 (#sc-130411, Santa cruz, TX, USA), anti-keratin 5 (KRT5; 1:200 dilutions; #905504, BioLegend, CA, USA) and anti-keratin 7 (KRT7; #4465, Cell Signaling Technology), anti-ARL13B (#17711-1-AP, Proteintech, IL, USA) and anti-acetylated α-tubulin (Ac-Tub; T7451, Sigma, CA, USA), DAPI (D9542, Sigma), Western blotting_anti- phosphorylated Met (pMet;#3135, Cell Signaling Technology), Met (#4560, Cell Signaling Technology), phosphorylated EGFR (pEGFR; #2234, Cell Signaling Technology), EGFR (#2232, Cell Signaling Technology), phosphorylated Akt (pAkt; #9271S, Cell Signaling Technology), Akt (#9272S, Cell Signaling Technology), phosphorylated Mek (pMek; #9121S, Cell Signaling Technology), Mek (#9122S, Cell Signaling Technology), phosphorylated Erk 1/2 (pErk;#9101S, Cell Signaling Technology), Erk 1/2 (Erk;#SC-135900, Santa Cruz) and GAPDH (#SC-32233, Santa Cruz). Secondary antibodies: Histology and imaging_#AI-2000, #AI-1000, Vector laboratories, CA, USA. Immunofluorescence_Alexa Fluor 488- (# A-11029, Invitrogen) and 594-conjugated secondary antibodies (# A-11037, Invitrogen). Western blotting_HRP-conjugated goat anti-rabbit (#ADI-SAB-300-J) or anti-mouse (#BML-SA204-0100) IgG secondary antibodies (Enzo Life Sciences, Inc., NY, USA) |
| Validation | Primary antibodies: histology and dilutionsimaging_anti-napsin A (1:200 dilutions, anti-mouse), anti-TTF-1 (1:200 dilutions, anti-mouse), anti-cytokeratin 7 (CK7; 1:400 dilutions, anti-mouse), anti-p63 (1:200 dilutions, anti-mouse), anti-cytokeratin 5/6 (CK5/6; 1:200 dilutions, anti-mouse), anti-CD133 (1:200 dilutions, anti-rabbit), anti-PD-L1 (1:200 dilution, anti-rabbit), and anti-c-Met (1:400 dilutions, anti-mouse). Immunofluorescence_anti-pancytokeratin (Pan CK; 1:1000 dilutions, anti-mouse), anti-p63 (1:1000 dilutions, anti-rabbit), anti-mucin 1 (MUC1; 1:500, anti-rabbit), anti-CC10 (1:200 dilutions, anti-mouse), anti-keratin 5 (KRT5; 1:200 dilutions, anti-rabbit) and anti-keratin 7 (KRT7; 1:200 dilutions, anti-rabbit), anti-ARL13B (1:1,000 dilutions, anti-rabbit) and |

anti-acetylated α-tubulin (Ac-Tub; 1:1,000 dilutions, anti-mouse), DAPI (1:2000 dilutions), Western blotting_anti- phosphorylated Met (pMet; 1:1000 dilutions, anti-rabbit), Met (1:1000 dilutions, anti-rabbit), phosphorylated EGFR (pEGFR; 1:1000 dilutions, anti-rabbit), EGFR (1:1000 dilutions, anti-rabbit), phosphorylated Akt (pAkt; 1:1000 dilutions, anti-rabbit), Akt (1:1000 dilutions, anti-rabbit), phosphorylated Mek (pMek; 1:1000 dilutions, anti-rabbit), Mek (1:1000 dilutions, anti-rabbit), phosphorylated Erk 1/2 (pErk; 1:1000 dilutions, anti-rabbit), Erk 1/2 (Erk; 1:1000 dilutions, anti-mouse) and GAPDH (1:1000 dilutions, anti-mouse). Secondary antibodies: Histology and imaging_Horse Anti Mouse IgG (#AI-2000), Goat Anti Rabbit IgG (#AI-1000) for 1:5000 dilutions. Immunofluorescence_Alexa Fluor 488- conjugated anti-mouse- (1:1000 dilutions) and 594-conjugated anti-rabbit-secondary antibodies (1:1000 dilutions). Western blotting_HRP-conjugated goat anti-rabbit (#ADI-SAB-300-J) or anti-mouse (#BML-SA204-0100) IgG secondary antibodies (1:5000). All antibodies used in the study are validated for species by manufacturer.

## Animals and other organisms

Policy information about studies involving animals; ARRIVE guidelines recommended for reporting animal research

| | |
|---|---|
| Laboratory animals | NOD scid gamma mice (NOD.Cg-Prkdcscid Il2rgtm1Wjl/SzJ), male, 6 weeks old from Jackson lab. |
| Wild animals | This study did not involve wild animals. |
| Field-collected samples | This study did not involve samples collected from the field. |
| Ethics oversight | The entire experimental protocol was conducted in compliance with the institutional guidelines and approved by the institutional animal care and use committee (IACUC) of the Asan Institute for Life Sciences, Asan Medical Center, Korea. |

Note that full information on the approval of the study protocol must also be provided in the manuscript.

## Human research participants

Policy information about studies involving human research participants

| | |
|---|---|
| Population characteristics | We included detailed population characteristics in the Table 1 - 3. |
| Recruitment | We recruited participants who had a surgery in Asan Medical Center with patients' consent. |
| Ethics oversight | The research protocol was approved by the Ethics Committee of Asan Medical Center (Seoul, Korea). The entire experimental protocol was conducted in compliance with the institutional guidelines. |

Note that full information on the approval of the study protocol must also be provided in the manuscript.
